# Association Between Left Atrial Enlargement and Atrial Fibrillation in Elderly Patients with Left Ventricular Diastolic Dysfunction

**DOI:** 10.3390/jcm15197480

**Published:** 2026-09-25

**Authors:** Ameen Abdulhasan Al-Alwany, Faris Kadhim, Mohammed Imad Salim, Mustafa Majid, Haitham Nabeel, Umniah Khajori, Matteo Luigi Giuseppe Leoni, Giustino Varrassi

**Affiliations:** 1 College of Medicine, University of Baghdad, Baghdad 10047, Iraqmostafa.maged1600d@comed.uobaghdad.edu.iq (M.M.); omniya.rafed1700a@comed.uobaghdad.edu.iq (U.K.); 2 Department of Medical and Surgical Sciences and Translational Medicine, Sapienza University of Roma, 00135 Roma, Italy; matteolg.leoni@gmail.com; 3 Research Department, Fondazione Paolo Procacci, 00193 Roma, Italy

**Keywords:** atrial fibrillation, left ventricular diastolic dysfunction, left atrial enlargement, left atrial volume index, echocardiography, elderly patients, atrial remodeling

## Abstract

**Background:** Left ventricular diastolic dysfunction (LVDD) is associated with chronically elevated left ventricular filling pressures that promote left atrial (LA) remodeling and increase the risk of atrial fibrillation (AF). Because AF is frequently asymptomatic during its early stages, identifying echocardiographic markers that facilitate early risk stratification is of considerable clinical importance. This study investigated the association between LA enlargement and AF in elderly patients with LVDD using conventional echocardiographic LA size parameters. **Methods:** This prospective observational study enrolled 100 consecutive patients aged ≥ 60 years with echocardiographically confirmed LVDD and no documented history of AF between December 2023 and May 2024. Comprehensive transthoracic echocardiography was performed to assess LA diameter, biplane LA volume using Simpson’s method, LA volume by the area–length method, and indexed LA volume (LAVI). The presence of AF was determined by standard 12-lead electrocardiography and rhythm assessment. Echocardiographic parameters were compared between patients with and without AF using appropriate statistical analyses. **Results:** The study population had a mean age of 68.37 ± 6.31 years, and 52% were male. AF was identified in 15% of participants, while mild LA enlargement was observed in 18%. Among patients with LA enlargement, 72.2% had AF compared with only 2.4% of patients with a normal LA size (*p* < 0.001). Patients with AF demonstrated significantly greater biplane LA volume (69.10 ± 9.61 vs. 52.21 ± 6.28 mL), LA volume measured by the area–length method (71.42 ± 9.51 vs. 54.03 ± 6.53 mL), and indexed LA volume (37.33 ± 3.81 vs. 28.87 ± 3.65 mL/m^2^) than those without AF (all *p* < 0.001). **Conclusions:** Left atrial enlargement measured using echocardiographic assessment, particularly indexed LA volume, is strongly associated with atrial fibrillation in elderly patients with left ventricular diastolic dysfunction.

## 1. Introduction

Atrial fibrillation (AF) is the most frequently encountered sustained cardiac arrhythmia and remains one of the leading causes of cardiovascular morbidity in older adults. As life expectancy continues to increase worldwide, the number of individuals affected by AF is expected to rise substantially, making it an increasingly important public health issue. Epidemiological studies indicate that the probability of developing AF during a lifetime approaches one-third in adults, with incidence increasing sharply after the sixth decade of life. Beyond its growing prevalence, AF carries important clinical consequences, including an increased risk of ischemic stroke (especially CHA2DS2-VA score 2 or more) heart failure, cognitive decline, repeated hospital admissions, and premature death. These complications place a considerable burden on both patients and healthcare systems, underscoring the importance of identifying individuals at high risk before clinically overt arrhythmia develops [1,2,3].

The mechanisms responsible for AF are diverse and involve a combination of structural remodeling, electrophysiological alterations, and neurohormonal activation within the atrial myocardium. Aging contributes significantly to these processes by promoting myocardial fibrosis, chronic inflammation, oxidative stress, and changes in atrial conduction properties. These age-related changes are often compounded by common cardiovascular disorders such as hypertension, diabetes mellitus, coronary artery disease, obesity, and valvular heart disease, all of which increase atrial pressure or volume loading and accelerate atrial remodeling. In addition, AF can occur merely due to atrial ectopic defects like in potassium channel gain mutation in familial AF, without major structural dysfunction [4,5,6].

Left ventricular diastolic dysfunction (LVDD) has gained increasing recognition as an important contributor to this pathological process. Impaired ventricular relaxation and reduced myocardial compliance result in elevated left ventricular filling pressures despite preserved systolic function. Persistent transmission of these elevated pressures to the left atrium exposes the atrial wall to chronic mechanical stress, leading to progressive enlargement, fibrosis, and deterioration of atrial mechanical performance [4,7,8].

Growing evidence suggests that left atrial remodeling is more than a secondary consequence of elevated filling pressures; rather, it represents an intermediate stage linking ventricular diastolic dysfunction with clinically significant atrial arrhythmias. Structural changes within the atrium include chamber dilatation, expansion of the extracellular matrix, and replacement fibrosis, accompanied by inflammatory activation and neurohormonal signaling, particularly through the renin–angiotensin–aldosterone system [4,7,8].

Echocardiography remains the cornerstone of non-invasive assessment of left atrial structure and function in routine clinical practice. Although linear left atrial diameter has historically been used to estimate atrial size, current echocardiographic guidelines emphasize volumetric assessment because atrial remodeling occurs in three dimensions and cannot be adequately characterized by a single linear measurement. Left atrial volume (LAV), especially when indexed to body surface area (LAVI), has therefore become the preferred parameter for evaluating chronic atrial remodeling. Numerous investigations have shown that an increased LAVI is associated with future AF, stroke, heart failure with preserved ejection fraction, and adverse cardiovascular outcomes, supporting its role as an integrated marker of long-standing elevation in left ventricular filling pressure [4,7,9,10].

Recent advances in cardiac imaging, particularly speckle-tracking echocardiography and assessment of left atrial strain, have enabled the detection of subtle atrial dysfunction before structural enlargement becomes apparent. Despite their promising diagnostic value, these techniques require specialized equipment and expertise and are not routinely available in many healthcare settings, particularly in resource-limited regions [4,11].

Although previous studies have consistently linked left atrial enlargement with AF, several questions remain unanswered. Many published investigations have examined heterogeneous patient populations with mixed cardiovascular conditions, making it difficult to define the specific relationship between atrial remodeling and AF in elderly individuals with isolated LVDD. Furthermore, prospective data from Middle Eastern populations are limited, despite regional differences in demographic characteristics and cardiovascular risk profiles. Addressing these gaps may improve patient selection for rhythm surveillance and facilitate earlier recognition of individuals at greatest risk for developing AF [11,12].

The present prospective observational study was designed to investigate the relationship between left atrial enlargement (left atrial diameter, left atrial volume, and indexed left atrial volume) and atrial fibrillation in elderly patients with echocardiographically confirmed LVDD. We hypothesized that greater degrees of left atrial remodeling would be associated with a higher prevalence of atrial fibrillation, supporting the incorporation of comprehensive left atrial assessment into the routine evaluation of elderly patients with diastolic dysfunction.

## 2. Materials and Methods

### 2.1. Study Design and Setting

This prospective observational study was conducted between December 2023 and May 2024 at Baghdad Teaching Hospital/The Iraqi Center for Cardiovascular Diseases, Baghdad, Iraq. The primary objective was to investigate the relationship between left atrial (LA) enlargement and the presence of atrial fibrillation (AF) among elderly patients with left ventricular diastolic dysfunction (LVDD). Consecutive patients fulfilling the eligibility criteria were prospectively enrolled during the study period to reduce selection bias and improve the representativeness of the study population. The study was designed and reported in accordance with the Strengthening the Reporting of Observational Studies in Epidemiology (STROBE) statement for observational studies [13].

### 2.2. Study Population

Patients aged 60 years or older who underwent comprehensive transthoracic echocardiography and demonstrated evidence of LVDD were screened for eligibility. A total of 100 consecutive patients meeting the inclusion criteria were included in the final analysis.

#### 2.2.1. Inclusion Criteria

Patients were eligible for enrollment if they fulfilled **all** the following criteria:

- Age ≥ 60 years.
- Echocardiographic diagnosis of left ventricular diastolic dysfunction (Grade I or Grade II) or elevated LA pressure in newly enrolled AF patients according to the recommendations of the American Society of Echocardiography and the European Association of Cardiovascular Imaging. To avoid incorporation bias, LAVI assessment criteria were excluded from the DD grading [14].
- Ability to provide verbal informed consent.
- No previously documented history of atrial fibrillation.

#### 2.2.2. Exclusion Criteria

Patients were excluded if **any** of the following conditions were present:

- Age below 60 years.
- Previous diagnosis of atrial fibrillation or atrial flutter.
- Documented ischemic heart disease.
- Moderate-to-severe mitral valve disease or previous mitral valve surgery.
- Poor echocardiographic image quality preventing accurate assessment of left atrial dimensions.

### 2.3. Clinical Assessment

At enrollment, all participants underwent a standardized clinical evaluation performed by experienced cardiologists. Demographic information, cardiovascular history, and relevant clinical characteristics were recorded using a structured data collection form. Age, sex, and cardiovascular risk factors were documented for all participants before echocardiographic examination. Resting blood pressure and heart rate were measured after an adequate period of rest using standard clinical procedures.

### 2.4. Echocardiographic Examination

Comprehensive transthoracic echocardiography was performed using a commercially available ultrasound system (Vivid E9, GE Healthcare, Horten, Norway) equipped with a phased-array transducer. All examinations were performed by experienced echocardiographers according to the recommendations of the American Society of Echocardiography (ASE) and the European Association of Cardiovascular Imaging (EACVI) [14].

The investigator (echocardiographic specialist) examined patients in the left lateral decubitus position, and standard parasternal long-axis, parasternal short-axis, apical four-chamber, and apical two-chamber views were acquired. Two-dimensional, M-mode, pulsed-wave Doppler, continuous-wave Doppler, color Doppler, and tissue Doppler imaging were obtained as clinically indicated. For sinus rhythm, a reading was taken from single beat, for AF rhythm, average values of 5 beats were taken. The investigator was blinded of the original rhythm of the patients.

Left atrial size was assessed using complementary structural measurements to provide a comprehensive evaluation of atrial remodeling. The following parameters were measured:

- Left atrial anteroposterior diameter obtained from the parasternal long-axis view at end-systole.
- Left atrial volume measured using the biplane method of disks (modified Simpson’s method) from the apical four- and two-chamber views.
- Left atrial volume calculated using the area–length method.
- Left atrial volume index (LAVI), calculated by dividing left atrial volume by body surface area and expressed in mL/m^2^.

A LAVI less than 34 indicated a normal atrium. A LAVI of 34 to 41 mL/m^2^ indicated mild enlargement [15].

Because volumetric assessment more accurately reflects three-dimensional atrial remodeling than linear measurements, LAVI was considered the principal echocardiographic marker of chronic left atrial remodeling [14,16].

Left ventricular diastolic function was evaluated according to current ASE/EACVI recommendations using mitral inflow velocities (E and A waves), tissue Doppler-derived mitral annular early diastolic velocity (e′), E/e′ ratio, tricuspid regurgitation velocity, and indexed left atrial volume. Patients were classified as having Grade I or Grade II diastolic dysfunction according to established guideline criteria. In AF patients, diastolic dysfunction (left atrial filling pressure) was estimated from 5 average beats according to ASE (deceleration time, septal E/e′, TR velocity and mitral E flow velocity). All patients had a heart rate below 100 beats per minute [14].

### 2.5. Assessment of Atrial Fibrillation

The primary outcome of the study was the presence of atrial fibrillation at the time of clinical evaluation. All participants underwent a standard 12-lead electrocardiogram (ECG) on the day of echocardiographic examination. AF was diagnosed according to the 2024 European Society of Cardiology Guidelines, defined as an irregularly irregular cardiac rhythm without identifiable P waves on a standard ECG recording [17]. Available rhythm documentation was reviewed to confirm the diagnosis whenever necessary. Patients with a documented history of AF before study enrollment were excluded.

### 2.6. Study Outcomes

The primary objective was to determine the association between echocardiographic evidence of left atrial enlargement and the presence of atrial fibrillation in elderly patients with LVDD.

Secondary objectives included comparison of the following echocardiographic measurements between patients with and without AF:

- Biplane left atrial volume measured using Simpson’s method.
- Left atrial volume measured using the area–length method.
- Indexed left atrial volume (LAVI).

### 2.7. Statistical Analysis

Statistical analyses were performed using IBM SPSS Statistics version 21.0 (IBM Corp., Armonk, NY, USA). Normally distributed variables are presented as mean ± standard deviation (SD), whereas categorical variables are expressed as frequencies and percentages.

Sample size was determined by the convenient consecutive enrollment of *n* = 100 eligible patients during the operational study window.

Comparisons between patients with and without atrial fibrillation were performed using the independent sample Student’s *t*-test for continuous variables and the Chi-square test or Fisher’s exact test for categorical variables. R-studio (logistif and car packages) was used to elaborate firth regression to reduce small-sample bias; the assumptions of independent observations and linearity of continuous predictors in the logit using Box-Tidwell tests were assessed. Multicollinearity among the predictor variables was evaluated using variance inflation factors (VIFs) (less than 2 is considered acceptable). Potential influential observations and outliers were also assessed.

Effect estimates are reported as Adjusted Odds Ratios with corresponding 95% Profile Likelihood Confidence Intervals and two-tailed *p*-values, with statistical significance set at *p*-values less than 0.05.

### 2.8. Ethical Approval

The study protocol was approved by the Research Ethics Committee of the College of Medicine, University of Baghdad, before patient recruitment. Verbal informed consent was obtained from all participants prior to enrollment. All procedures were conducted in accordance with the ethical principles of the Declaration of Helsinki for research involving human subjects [17]. Patient confidentiality was maintained throughout the study by anonymizing all data collected before statistical analysis.

## 3. Results

### 3.1. Study Population

During the study period, 100 consecutive elderly patients with echocardiographically confirmed left ventricular diastolic dysfunction fulfilled the eligibility criteria and were included in the final analysis.

The study cohort comprised 52 men (52%) and 48 women (48%), with a mean age of 68.37 ± 6.31 years. All participants completed the planned clinical and echocardiographic evaluation, and no patient was excluded because of incomplete imaging or missing data (Figure 1). Atrial fibrillation was identified in 15 patients (15%), whereas the remaining 85 patients (85%) were in sinus rhythm at the time of assessment. Mild left atrial enlargement was observed in 18 patients (18%), while 82 patients (82%) had normal left atrial dimensions according to echocardiographic criteria. Figure 1 shows the sample selection process.

### 3.2. Association Between Left Atrial Enlargement and Atrial Fibrillation

A significant association was observed between left atrial enlargement and the presence of atrial fibrillation. Among patients with mild LA enlargement, 13 of 18 patients (72.2%) had AF, whereas only 2 of 82 patients (2.4%) with a normal LA size were diagnosed with AF.

Conversely, 80 of 82 patients (97.6%) with a normal left atrial size remained in sinus rhythm compared with only 5 of 18 patients (27.8%) with enlarged atria. This difference was highly statistically significant (*p* < 0.001), indicating a strong association between structural left atrial remodeling and the presence of atrial fibrillation.

### 3.3. Echocardiographic Characteristics According to Rhythm Status

Patients with atrial fibrillation demonstrated significantly larger left atrial dimensions than those without AF (Table 1 and Table 2). Mean left atrial volume measured using the biplane Simpson method was significantly higher in the AF group (69.10 ± 9.61 mL) compared with patients in sinus rhythm (52.21 ± 6.28 mL, *p* < 0.001). Similarly, left atrial volume calculated using the area–length method was significantly greater in patients with AF (71.42 ± 9.51 mL) than in those without AF (54.03 ± 6.53 mL, *p* < 0.001).

The indexed left atrial volume (LAVI) also differed significantly between the two groups, with AF patients demonstrating a mean value of 37.33 ± 3.81 mL/m^2^, compared with 28.87 ± 3.65 mL/m^2^ among patients in sinus rhythm (*p* < 0.001). Overall, all volumetric measurements consistently demonstrated greater atrial remodeling in patients with AF.

### 3.4. Cofactor-Based Analysis

When featuring firth regression for the low prevalence of AF in our study. Only LAVI showed a significant association with AF. Table 3 presents the results.

## 4. Discussion

The present prospective observational study demonstrated a significant association between left atrial (LA) enlargement and the presence of atrial fibrillation (AF) in elderly patients with left ventricular diastolic dysfunction (LVDD). Patients with enlarged left atria exhibited a markedly higher prevalence of AF than those with normal atrial dimensions. Moreover, all volumetric echocardiographic indices, including biplane LA volume, area–length LA volume, and indexed left atrial volume (LAVI), were significantly greater in patients with AF. These findings suggest that structural remodeling of the left atrium is closely associated with AF in elderly individuals with impaired diastolic function and further support the clinical importance of routine echocardiographic assessment of LA size in this high-risk population.

The association observed in the present study is consistent with the established pathophysiological relationship between LVDD and atrial remodeling. Impaired ventricular relaxation and increased myocardial stiffness elevate left ventricular filling pressures, which are chronically transmitted to the left atrium. Sustained pressure overload promotes atrial dilatation, extracellular matrix expansion, myocardial fibrosis, and progressive deterioration of atrial compliance. These structural alterations are accompanied by electrical remodeling characterized by slowed conduction velocity, heterogeneous refractoriness, and increased susceptibility to re-entry mechanisms, thereby facilitating the development and maintenance of AF [4,5,8,18]. Consequently, LA enlargement should be regarded not merely as an anatomical finding but as a structural manifestation of chronic diastolic dysfunction and atrial remodeling.

Our findings are consistent with previous investigations demonstrating that volumetric assessment of the left atrium is more informative than conventional linear measurements. Thomas et al. emphasized that left atrial volume reflects cumulative exposure to elevated filling pressures and provides a more accurate representation of chronic atrial remodeling than left atrial diameter because atrial enlargement occurs asymmetrically in three dimensions [4]. Likewise, Bossard et al. reported that increased left atrial volume is independently associated with atrial fibrillation and represents an important indicator of structural atrial remodeling [10]. The significantly greater biplane LA volume and area–length LA volume observed in our patients with AF further support these observations and reinforce the value of volumetric assessment during routine echocardiographic examination.

Among the evaluated echocardiographic parameters, indexed left atrial volume demonstrated the strongest association with AF. Unlike absolute LA volume, LAVI accounts for differences in body size and therefore provides a standardized assessment of atrial remodeling across diverse patient populations. Similarly, Pawar et al. demonstrated a significant relationship between increased LAVI, LVDD, and cardiovascular risk factors, supporting its role as an integrated marker of chronic diastolic burden [9]. In our cohort, patients with AF exhibited substantially higher LAVI values than those in sinus rhythm, further supporting its clinical relevance in elderly patients with LVDD.

Recent advances in cardiovascular imaging indicate that impairment of left atrial function may precede detectable structural enlargement. Speckle-tracking echocardiography and assessment of left atrial reservoir strain have emerged as sensitive methods for identifying early atrial dysfunction before conventional echocardiographic abnormalities become apparent [11]. Nevertheless, these techniques remain unavailable in many routine clinical settings, particularly in resource-limited healthcare systems. Therefore, conventional echocardiographic parameters such as LA volume and LAVI continue to represent practical, reproducible, and widely accessible tools for risk stratification in everyday clinical practice. The present findings suggest that careful assessment of these readily available measurements may improve the identification of elderly patients with LVDD who are more likely to have concomitant AF [19].

The relationship between atrial remodeling and AF was observed in both male and female participants. Although absolute indexed LA volumes were slightly greater in male patients, the overall association between LA enlargement and AF remained consistent across both sexes. These observations suggest that the structural consequences of chronic diastolic dysfunction affect both men and women similarly and support previous evidence indicating that atrial remodeling is primarily driven by hemodynamic burden rather than sex alone [4,20,21,22].

The clinical implications of the present findings deserve consideration. AF frequently remains clinically silent until the occurrence of stroke or heart failure decompensation. Consequently, early recognition of patients at increased risk is essential for timely diagnosis and management. Comprehensive assessment of LA size during routine transthoracic echocardiography requires no additional imaging equipment or substantial increase in examination time, making it an attractive strategy for routine cardiovascular evaluation. Identification of significant atrial remodeling may prompt closer rhythm surveillance, prolonged electrocardiographic monitoring, and earlier consideration of preventive therapeutic strategies in elderly patients with LVDD [1].

Several limitations should be acknowledged. First, this was a single-center observational study with a relatively modest sample size, which may limit the generalizability of the findings. Second, because AF and echocardiographic measurements were assessed at a single time point, the study demonstrates association rather than causation and cannot determine whether LA enlargement precedes the development of AF. Third, continuous ambulatory rhythm monitoring was not systematically performed, and asymptomatic paroxysmal AF may therefore have been underestimated. Finally, advanced imaging modalities such as left atrial strain analysis and cardiac magnetic resonance imaging were not available, preventing more detailed characterization of atrial remodeling.

The present study has several strengths. Consecutive patient enrollment reduced the likelihood of selection bias, while comprehensive echocardiographic assessment included multiple complementary measures of LA size rather than relying solely on linear diameter. Furthermore, the study specifically evaluated an elderly population with LVDD, a clinically relevant group at increased risk for atrial remodeling and AF that remains underrepresented in the available literature from the Middle East.

Future multicenter studies including larger populations are required to validate these findings. Incorporation of advanced echocardiographic techniques, cardiac magnetic resonance imaging, circulating biomarkers of myocardial fibrosis, and artificial intelligence-assisted image analysis may further improve identification of patients at increased risk for AF. In addition, longitudinal studies are warranted to determine whether progressive increases in LAVI predict the subsequent development of AF and whether earlier intervention in patients with significant atrial remodeling improves long-term cardiovascular outcomes [11,22,23,24,25].

## 5. Conclusions

The present study of older adults with left ventricular diastolic dysfunction, left atrial enlargement and higher left atrial volume measurements were significantly associated with atrial fibrillation detected at the time of clinical assessment. Participants with atrial fibrillation had a greater biplane left atrial volume, area–length left atrial volume and indexed left atrial volume than those in sinus rhythm.

These findings support the clinical relevance of conventional echocardiographic assessment of left atrial size in older patients with diastolic dysfunction. In particular, left atrial volume index may help identify patients in whom closer rhythm evaluation could be considered. However, because left atrial dimensions and cardiac rhythm were assessed concurrently, the present study cannot establish whether left atrial enlargement preceded atrial fibrillation or whether it can predict the future development of the arrhythmia. Reverse causation also cannot be excluded, as atrial fibrillation itself may contribute to left atrial remodeling.

Larger longitudinal studies using systematic ambulatory rhythm monitoring, standardized echocardiographic protocols, and adjustment for relevant cardiovascular confounders are needed to determine whether left atrial volume independently predicts incident atrial fibrillation in this population.

## Figures and Tables

**Figure 1:**
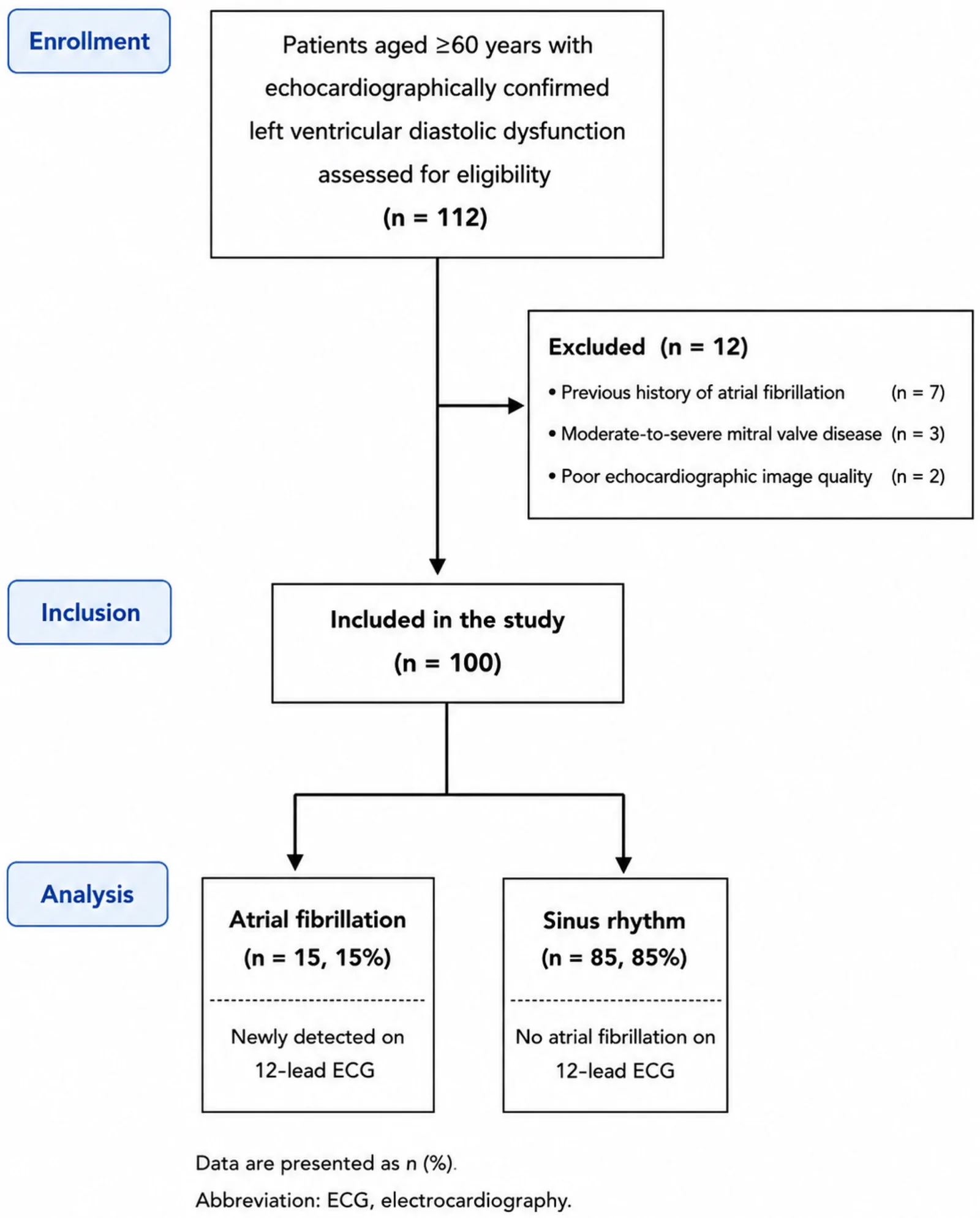
Study Flow Diagram.

**Table 1:** Distribution of Atrial Fibrillation According to Left Atrial Size Stratification.

LA Size Grading	AF Patients (*n* = 15)	Sinus Rhythm Patients (*n* = 85)	Total (*n* = 100)	*p*-Value
Mild Enlargement	13 (72.2%)	5 (27.8%)	18	<0.001
Normal	2 (2.4%)	80 (97.6%)	82	

**Table 2:** Mean Echocardiographic Parameters in Patients With and Without AF.

Parameter	AF (*n* = 15)	No AF (*n* = 85)	*p*-Value
Biplane LA Volume (Simpson)	69.10 ± 9.61 mL	52.21 ± 6.28 mL	<0.001
LA Volume (Area-Length Method)	71.42 ± 9.51 mL	54.03 ± 6.53 mL	<0.001
Indexed LA Volume	37.33 ± 3.81 mL/m^2^	28.87 ± 3.65 mL/m^2^	<0.001

**Table 3:** Firth regression for AF variables among the patients.

Variable	Coefficient (β)	SE	Adjusted OR	95% Profile Likelihood CI	*p*-Value
LAVI	0.401	0.086	1.49 *	1.28–1.84	<0.001
Smoking	0.642	0.728	1.90	0.41–9.35	0.405
Gender	0.522	0.732	1.69	0.37–8.73	0.500
Age **	−0.042	0.061	0.96	0.83–1.09	0.520
Hypertension	−0.657	0.738	0.52	0.10–2.45	0.402

* per 1 mL/m^2^ increase in LAVI. ** per 1 year increase in age. Categorical variable was made into a binary variable (e.g., hypertension or not).

## Data Availability

The data supporting the findings of this study are available from the corresponding author upon reasonable request.

## Abbreviations

The following abbreviations are used in this manuscript:

AF	Atrial Fibrillation
AI	Artificial Intelligence
ASE	American Society of Echocardiography
ECG	Electrocardiogram
EACVI	European Association of Cardiovascular Imaging
ESC	European Society of Cardiology
HFpEF	Heart Failure with Preserved Ejection Fraction
LA	Left Atrium/Left Atrial
LAV	Left Atrial Volume
LAVI	Left Atrial Volume Index
LV	Left Ventricle/Left Ventricular
LVDD	Left Ventricular Diastolic Dysfunction
MRI	Magnetic Resonance Imaging
RAAS	Renin–Angiotensin–Aldosterone System
SD	Standard Deviation
STROBE	Strengthening the Reporting of Observational Studies in Epidemiology
TTE	Transthoracic Echocardiography
